# Supplementary material for: A fault-intrusion-tolerant system and deadline-aware algorithm for scheduling scientific workflow in the cloud
Source: PeerJ Comput Sci. 2021 Nov 2;7:e747. doi: 10.7717/peerj-cs.747 (PMC8576554; doi:10.7717/peerj-cs.747)
Supplement: Supplemental Information 6 [file peerj-cs-07-747-s006.zip › Myproject1.1/docs/org/cloudbus/cloudsim/InfoPacket.html]

InfoPacket


JavaScript is disabled on your browser.


- Overview
- Package
- Class
- Use
- Tree
- Deprecated
- Index
- Help

- Prev Class
- Next Class

- Frames
- No Frames

- All Classes

- Summary:
- Nested |
- Field |
- Constr |
- Method

- Detail:
- Field |
- Constr |
- Method


org.cloudbus.cloudsim

## Class InfoPacket

- java.lang.Object
- - org.cloudbus.cloudsim.InfoPacket

- All Implemented Interfaces:
  :   Packet

  ---

    

  ```
  public class InfoPacket
  extends java.lang.Object
  implements Packet
  ```

  InfoPacket class can be used to gather information from the network layer. An InfoPacket
  traverses the network topology similar to a `gridsim.net.NetPacket`, but it collects
  information like bandwidths, and Round Trip Time etc. It is the equivalent of ICMP in physical
  networks.

  You can set all the parameters to an InfoPacket that can be applied to a NetPacket. So if you
  want to find out the kind of information that a particular type of NetPacket is experiencing, set
  the size and network class of an InfoPacket to the same as the NetPacket, and send it to the same
  destination from the same source.

  Since:
  :   CloudSim Toolkit 1.0

- - ### Constructor Summary

    Constructors

    Constructor and Description

    `InfoPacket(java.lang.String name,
    int packetID,
    long size,
    int srcID,
    int destID,
    int netServiceType)`

    Constructs a new Information packet.
  - ### Method Summary

    Methods

    | Modifier and Type | Method and Description |
    | --- | --- |
    | `void` | `addBaudRate(double baudRate)` Every entity that the InfoPacket traverses should add the baud rate of the link on which this packet will be sent out next. |
    | `void` | `addEntryTime(double time)` This method should be called by routers and other entities when this InfoPacket reaches them along with the current simulation time. |
    | `void` | `addExitTime(double time)` This method should be called by routers and other entities when this InfoPacket is leaving them. |
    | `void` | `addHop(int id)` This method should be called by network entities that count as hops, for e.g. |
    | `double` | `getBaudRate()` Returns the bottleneck bandwidth between the source and the destination. |
    | `int` | `getDestId()` Gets the id of the entity to which the packet is destined. |
    | `java.lang.Object[]` | `getDetailBaudRate()` Returns the list of all the bandwidths that this packet has traversed. |
    | `java.lang.Object[]` | `getDetailEntryTimes()` Returns the list of all entry time that this packet has traversed. |
    | `java.lang.Object[]` | `getDetailExitTimes()` Returns the list of all exit time that this packet has traversed. |
    | `java.lang.Object[]` | `getDetailHops()` Returns the list of all the hops that this packet has traversed. |
    | `int` | `getId()` Returns the ID of this packet. |
    | `int` | `getLast()` Gets an entity ID from the last hop that this packet has traversed. |
    | `int` | `getNetServiceType()` Gets the network service type of this packet. |
    | `int` | `getNumHop()` Returns the number of hops that this packet has traversed. |
    | `long` | `getOriginalPingSize()` Gets original size of ping request. |
    | `long` | `getSize()` Gets the size of this packet. |
    | `int` | `getSrcId()` Gets the id of the entity that sent out this packet. |
    | `int` | `getTag()` Gets this packet tag. |
    | `double` | `getTotalResponseTime()` Gets the total time that this packet has spent in the network. |
    | `void` | `setDestId(int id)` Sets the destination ID for this packet. |
    | `void` | `setLast(int last)` Sets an entity ID from the last hop that this packet has traversed. |
    | `void` | `setNetServiceType(int netServiceType)` Sets the network service type of this packet. |
    | `void` | `setOriginalPingSize(long size)` Sets original size of ping request. |
    | `boolean` | `setSize(long size)` Sets the size of this packet. |
    | `boolean` | `setTag(int tag)` Sets the tag of this packet. |
    | `java.lang.String` | `toString()` Returns a human-readable information of this packet. |

    - ### Methods inherited from class java.lang.Object

      `clone, equals, finalize, getClass, hashCode, notify, notifyAll, wait, wait, wait`

- - ### Constructor Detail


    - #### InfoPacket

      ```
      public InfoPacket(java.lang.String name,
                int packetID,
                long size,
                int srcID,
                int destID,
                int netServiceType)
      ```

      Constructs a new Information packet.

      Parameters:
      :   `name` - Name of this packet
      :   `packetID` - The ID of this packet
      :   `size` - size of the packet
      :   `srcID` - The ID of the entity that sends out this packet
      :   `destID` - The ID of the entity to which this packet is destined
      :   `netServiceType` - the class of traffic this packet belongs to
  - ### Method Detail


    - #### getId

      ```
      public int getId()
      ```

      Returns the ID of this packet.

      **Specified by:**
      :   `getId` in interface `Packet`

      Returns:
      :   packet ID


    - #### setOriginalPingSize

      ```
      public void setOriginalPingSize(long size)
      ```

      Sets original size of ping request.

      Parameters:
      :   `size` - ping data size (in bytes)


    - #### getOriginalPingSize

      ```
      public long getOriginalPingSize()
      ```

      Gets original size of ping request.

      Returns:
      :   original size


    - #### toString

      ```
      public java.lang.String toString()
      ```

      Returns a human-readable information of this packet.

      **Specified by:**
      :   `toString` in interface `Packet`

      **Overrides:**
      :   `toString` in class `java.lang.Object`

      Returns:
      :   description of this packet


    - #### getSize

      ```
      public long getSize()
      ```

      Gets the size of this packet.

      **Specified by:**
      :   `getSize` in interface `Packet`

      Returns:
      :   size of the packet.


    - #### setSize

      ```
      public boolean setSize(long size)
      ```

      Sets the size of this packet.

      **Specified by:**
      :   `setSize` in interface `Packet`

      Parameters:
      :   `size` - size of the packet

      Returns:
      :   true if it is successful, false otherwise


    - #### getDestId

      ```
      public int getDestId()
      ```

      Gets the id of the entity to which the packet is destined.

      **Specified by:**
      :   `getDestId` in interface `Packet`

      Returns:
      :   the desination ID


    - #### getSrcId

      ```
      public int getSrcId()
      ```

      Gets the id of the entity that sent out this packet.

      **Specified by:**
      :   `getSrcId` in interface `Packet`

      Returns:
      :   the source ID


    - #### getNumHop

      ```
      public int getNumHop()
      ```

      Returns the number of hops that this packet has traversed. Since the packet takes a round
      trip, the same router may have been traversed twice.

      Returns:
      :   the number of hops this packet has traversed


    - #### getTotalResponseTime

      ```
      public double getTotalResponseTime()
      ```

      Gets the total time that this packet has spent in the network. This is basically the RTT.
      Dividing this by half should be the approximate latency.

      RTT is taken as the final entry time - first exit time.

      Returns:
      :   total round time


    - #### getBaudRate

      ```
      public double getBaudRate()
      ```

      Returns the bottleneck bandwidth between the source and the destination.

      Returns:
      :   the bottleneck bandwidth


    - #### addHop

      ```
      public void addHop(int id)
      ```

      This method should be called by network entities that count as hops, for e.g. Routers or
      CloudResources. It should not be called by links etc.

      Parameters:
      :   `id` - the id of the hop that this InfoPacket is traversing


    - #### addEntryTime

      ```
      public void addEntryTime(double time)
      ```

      This method should be called by routers and other entities when this InfoPacket reaches them
      along with the current simulation time.

      Parameters:
      :   `time` - current simulation time, use `gridsim.CloudSim#clock()` to obtain this


    - #### addExitTime

      ```
      public void addExitTime(double time)
      ```

      This method should be called by routers and other entities when this InfoPacket is leaving
      them. It should also supply the current simulation time.

      Parameters:
      :   `time` - current simulation time, use `gridsim.CloudSim#clock()` to obtain this


    - #### addBaudRate

      ```
      public void addBaudRate(double baudRate)
      ```

      Every entity that the InfoPacket traverses should add the baud rate of the link on which this
      packet will be sent out next.

      Parameters:
      :   `baudRate` - the entity's baud rate in bits/s


    - #### getDetailBaudRate

      ```
      public java.lang.Object[] getDetailBaudRate()
      ```

      Returns the list of all the bandwidths that this packet has traversed.

      Returns:
      :   a Double Array of links bandwidths


    - #### getDetailHops

      ```
      public java.lang.Object[] getDetailHops()
      ```

      Returns the list of all the hops that this packet has traversed.

      Returns:
      :   an Integer Array of hop ids


    - #### getDetailEntryTimes

      ```
      public java.lang.Object[] getDetailEntryTimes()
      ```

      Returns the list of all entry time that this packet has traversed.

      Returns:
      :   an Integer Array of entry time


    - #### getDetailExitTimes

      ```
      public java.lang.Object[] getDetailExitTimes()
      ```

      Returns the list of all exit time that this packet has traversed.

      Returns:
      :   an Integer Array of exit time


    - #### getLast

      ```
      public int getLast()
      ```

      Gets an entity ID from the last hop that this packet has traversed.

      **Specified by:**
      :   `getLast` in interface `Packet`

      Returns:
      :   an entity ID


    - #### setLast

      ```
      public void setLast(int last)
      ```

      Sets an entity ID from the last hop that this packet has traversed.

      **Specified by:**
      :   `setLast` in interface `Packet`

      Parameters:
      :   `last` - an entity ID from the last hop


    - #### getNetServiceType

      ```
      public int getNetServiceType()
      ```

      Gets the network service type of this packet.

      **Specified by:**
      :   `getNetServiceType` in interface `Packet`

      Returns:
      :   the network service type


    - #### setNetServiceType

      ```
      public void setNetServiceType(int netServiceType)
      ```

      Sets the network service type of this packet.

      **Specified by:**
      :   `setNetServiceType` in interface `Packet`

      Parameters:
      :   `netServiceType` - the packet's network service type


    - #### getTag

      ```
      public int getTag()
      ```

      Gets this packet tag.

      **Specified by:**
      :   `getTag` in interface `Packet`

      Returns:
      :   this packet tag


    - #### setTag

      ```
      public boolean setTag(int tag)
      ```

      Sets the tag of this packet.

      Parameters:
      :   `tag` - the packet's tag

      Returns:
      :   true if successful, false otherwise


    - #### setDestId

      ```
      public void setDestId(int id)
      ```

      Sets the destination ID for this packet.

      Parameters:
      :   `id` - this packet's destination ID


- Overview
- Package
- Class
- Use
- Tree
- Deprecated
- Index
- Help

- Prev Class
- Next Class

- Frames
- No Frames

- All Classes

- Summary:
- Nested |
- Field |
- Constr |
- Method

- Detail:
- Field |
- Constr |
- Method
